# Reliability of Transcallosal Inhibition in Healthy Adults

**DOI:** 10.3389/fnhum.2016.00681

**Published:** 2017-01-09

**Authors:** Melanie K. Fleming, Di J. Newham

**Affiliations:** Centre of Human and Aerospace Physiological Sciences, Faculty of Life Sciences and Medicine, King’s College LondonLondon, UK

**Keywords:** transcranial magnetic stimulation, transcallosal inhibition, interhemispheric inhibition, ipsilateral silent period, reliability

## Abstract

Transcallosal inhibition (TCI), assessed using transcranial magnetic stimulation, can provide insight into the neurophysiology of aging and of neurological disorders such as stroke. However, the reliability of TCI using the ipsilateral silent period (iSP) has not been formally assessed, despite its use in longitudinal studies. This study aimed to determine the reliability of iSP onset latency, duration and depth in healthy young and older adults. A sample of 18 younger (mean age 27.7 years, range: 19–42) and 13 older healthy adults (mean age 68.1 years, range: 58–79) attended four sessions whereby the iSP was measured from the first dorsal interosseous (FDI) muscle of each hand. 20 single pulse stimuli were delivered to each primary motor cortex at 80% maximum stimulator output while the participant maintained an isometric contraction of the ipsilateral FDI. The average onset latency, duration of the iSP, and depth of inhibition relative to baseline electromyography activity was calculated for each hand in each session. Intraclass correlation coefficients (ICCs) were calculated for all four sessions, or the first two sessions only. For iSP onset latency the reliability ranged from poor to good. For iSP duration there was moderate to good reliability (ICC > 0.6). Depth of inhibition demonstrated variation in reproducibility depending on which hand was assessed and whether two or four sessions were compared. Bland and Altman analyses showed wide limits of agreement between the first two sessions, particularly for iSP depth. However, there was no systematic pattern to the variability. These results indicate that although iSP duration is reliable in healthy adults, changes in longitudinal studies should be interpreted with caution, particularly for iSP depth. Future studies are needed to determine reliability in clinical populations.

## Introduction

Co-ordination of upper limb movement relies on communication between the two cerebral hemispheres. Each primary motor cortex (M1) interacts with the opposite one to prevent mirror movements and facilitate uni- and bi-manual movements (Hubers et al., 2008; Beaule et al., 2012). This is thought to be mediated at a cortical level (Ferbert et al., 1992) by pathways in the corpus callosum (Meyer et al., 1995; Boroojerdi et al., 1996). Interhemispheric inhibition can be assessed with transcranial magnetic stimulation (TMS) using either a paired pulse (dual coil) paradigm or the ipsilateral silent period (iSP; Ferbert et al., 1992; Reis et al., 2008; Perez and Cohen, 2009). With the paired pulse paradigm, the M1 of interest is conditioned by first applying a suprathreshold stimulus to the opposite M1 at a specific time interval and the reduction in motor evoked potential (MEP) amplitude is examined in comparison with a non-conditioned response. The iSP method assesses transcallosal inhibition (TCI) by applying a suprathreshold stimulus to M1 while the target muscle of the ipsilateral upper limb is activated voluntarily. This leads to a short interruption of the electromyography (EMG) activity. The iSP is absent or delayed in patients with lesions of the corpus callosum (Meyer et al., 1995, 1998) and altered in some patients after stroke (Boroojerdi et al., 1996; Shimizu et al., 2002; Murase et al., 2004). The iSP measure assesses the inhibition of volitional motor activity, making it a useful technique for examining the control of voluntary motor output (Beaule et al., 2012).

However, these two techniques are said to be complimentary rather than interchangeable (Chen et al., 2003). Both techniques assess TCI and are mediated by fibers passing through the corpus callosum, with increases in inhibition observed with increasing stimulus intensity up to ∼75% maximum stimulator output (MSO). However, only the iSP measure shows sensitivity to current direction (Chen et al., 2003). Chen et al. (2003) speculate that the two techniques involve different sets of callosal fibers or different neurons of the contralateral motor cortex.

Studies of TCI can provide insight into the neurophysiology of various conditions including healthy aging and neurological disorders such as stroke, and can be used as a marker of recovery of the balance in cortical excitability after interventions targeting the upper limb. Interhemispheric inhibition using the paired pulse technique has been found to have poor test–retest reliability in healthy adults (De Gennaro et al., 2003). However, to our knowledge the reliability of the iSP has not been assessed, despite its use in longitudinal studies. Older adults typically show delayed iSP onset latency and a lower magnitude of inhibition than younger adults (Davidson and Tremblay, 2013) as well as reduced bimanual coordination (Fling et al., 2011). It is therefore necessary to assess reliability in both of these populations. Thus, the aim of this study was to assess the reproducibility of iSP latency, depth, and duration in a group of healthy young and older adults.

## Materials and Methods

Healthy, community dwelling, adults (*n* = 31, mean age 45 years, range: 19–79 years) attended four sessions, whereby iSP measures were obtained from each hemisphere as part of a study examining the effect of transcranial direct current stimulation on motor sequence learning. Inclusion criteria were; aged > 18 years and right handed with no contraindications to TMS, such as epilepsy or seizures, cardiac pacemakers, metal implants above the neck or possible pregnancy. All participants were screened with a safety checklist and denied the presence of any neurological conditions or medications which could influence central nervous system excitability. Participants provided written informed consent and the study was approved by the local Research Ethics Committee. The iSP measures used for this analysis were obtained at the beginning of each session, prior to transcranial direct current stimulation. Sessions were at least 1 week apart [mean (SD) 11 (8) days]. The sample was divided into two groups; younger (<45 years) and older adults (>55 years), in order to determine whether the reliability of iSP latency, depth, or duration was affected by healthy aging. Group characteristics are provided in **Table 1**.

**Table 1:** Participant characteristics.

	Younger group (*n* = 18)	Older group (*n* = 13)
Age, years		
Mean (range)	27.7 (19–42)	68.1 (58–79)
Handedness, laterality quotient (%)		
Mean (range)	73.8 (42.9–100)	75.1 (33.3–100)
Sex, male		
n (%)	4 (22.2)	3 (23.1)

### Setup

Participants were seated with their hands resting on a pillow on their laps. TMS was delivered using a flat figure-of-eight coil (70 mm diameter) with a Magstim 200^2^ bistim stimulator (Magstim Company, UK). The coil was held on the scalp at ∼45° to induce a posterior to anterior current direction, approximately perpendicular to the central sulcus. The optimal position for evoking MEPs in the relaxed first dorsal interosseus (FDI) muscle was established in each session and marked with a water-soluble marker directly on the scalp to ensure consistent coil placement. EMG activity was recorded from each FDI using pairs of 13 mm Ag/AgCl Biotab electrodes (Unomedical, Ltd, UK) placed over the muscle in a belly tendon montage, following standard skin preparation techniques. Ground electrodes were placed over each ulnar styloid (23 mm Ag/AgCl Biotab electrode). The analog EMG data were pre-amplified 1000× (Digitimer, Ltd, Hertfordshire, UK) and bandpass filtered at 30–1000 Hz (Neurolog filter module, Digitimer, Ltd, UK) with a 50 Hz notch filter. Data were acquired at 2 kHz, A to D converted [1401, Cambridge Electronic Design, Ltd (CED), UK], recorded (Signal 4.07, CED, UK) and stored for off-line analysis.

### Transcallosal Inhibition

Transcallosal inhibition was assessed as the latency, depth and duration of the iSP, using a TMS intensity of 80% MSO, consistent with previous studies (Shimizu et al., 2002; Chen et al., 2003; Trompetto et al., 2004; Stinear et al., 2008, 2015; Spagnolo et al., 2013). Participants were instructed to activate the FDI muscle at ∼75% of their maximum voluntary effort, by pinching between the thumb and index finger or holding the index finger away from the middle finger (depending which produced the most consistent EMG activity for the individual). Twenty single pulse stimuli were delivered to the ipsilateral M1 with an interstimulus interval of 5–8 s and a short break (∼30 s) provided after every five stimuli to reduce the likelihood of fatigue. The hemisphere stimulated first was randomly chosen in each session.

**FIGURE 1:**
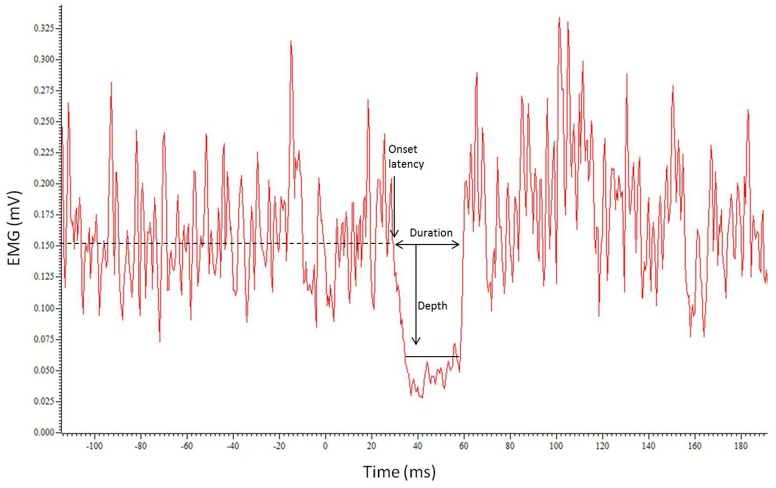
**Example trace showing the average of the 20 traces with the resulting silent period**. Horizontal dotted line indicates EMG level. Onset latency, duration of iSP and depth of inhibition are represented by arrows.

The latency, depth, and duration of the iSP were calculated using Signal 4.07 (CED, UK). Each trace was rectified then an average waveform constructed from the 20 traces (**Figure 1**). The pre-stimulus (baseline) root mean square (RMS) EMG was calculated for a 450 ms period ending 10 ms before the stimulus. The onset of the iSP (iSP latency) was calculated for the average waveform as the time where the EMG trace dropped below 75% of the pre-stimulus level. The depth of inhibition was calculated using the average waveform as the RMS EMG over the duration of the silent period expressed relative to baseline (% inhibition), using the formula: (RMS_BASELINE_ – RMS_ISP_)/RMS_BASELINE_ × 100. The iSP duration was calculated for each trace as the time from when the rectified EMG activity dropped below 75% of the pre-stimulus level to when it returned above 75%. This level of activity was chosen for onset and offset of the iSP to ensure a method of analysis that would be objective and robust, reducing the need for experimenter interpretation and minimizing bias. An average duration was calculated for each hemisphere in each session.

### Analysis

It has been suggested that reliability studies should utilize multiple analysis methods to enable a thorough investigation, including both intraclass correlation coefficients (ICCs) and Bland and Altman analyses (Rankin and Stokes, 1998). Therefore, both methods were utilized here. As there were four sessions, reliability of iSP latency, depth, and duration were assessed by comparing across all four sessions (ICCs) and also across the first two sessions (ICCs and Bland and Altman), for the younger and older groups separately. Each hemisphere was analyzed separately; “left FDI” refers to the TMS coil over the left M1 with the iSP recorded from the left FDI EMG and “right FDI” to the coil over the right M1 with the iSP recorded from the right FDI EMG.

Analysis was conducted using SPSS 21.0 (IBM, Inc.) and GraphPad Prism 5.02 (GraphPad Software, Inc., San Diego, CA, USA). Significance was set at *p* < 0.05.

#### Consistency Across Sessions

Intersession reliability was assessed using ICCs (type C, two way mixed effects model, average measures; SPSS 21.0). Values > 0.8 are considered to represent good reliability, 0.6–0.8 represents moderate reliability, and values below 0.6 indicate poor reliability. Bland and Altman plots were constructed to examine the pattern and spread of differences between the first two sessions against their average (Bland and Altman, 1986). The bias and 95% limits of agreement were calculated using GraphPad Prism 5.02.

#### Comparison between Sessions

Differences across the four sessions for baseline RMS EMG, iSP latency, depth, and duration were assessed using one way repeated measures analysis of variance (rmANOVA) if data were normally distributed and Friedman tests if not.

## Results

### Consistency Across Sessions

**FIGURE 2:**
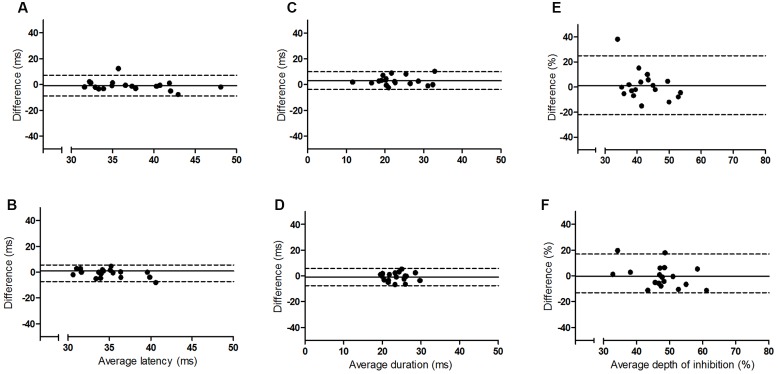
**Bland and Altman plots showing the difference between sessions one and two as a function of the average for the younger group**. Left panel **(A,B)** iSP latency, middle panel **(C,D)** iSP duration, right panel **(E,F)** iSP depth. Top row = left FDI, bottom row = right FDI. Solid line indicates mean difference (bias), dotted lines represent upper and lower 95% limits of agreement.

**FIGURE 3:**
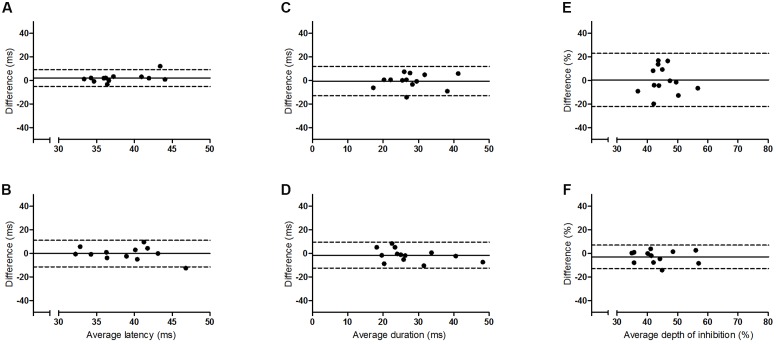
**Bland and Altman plots showing the difference between sessions one and two as a function of the average for the older group**. Left panel **(A,B)** iSP latency, middle panel **(C,D)** iSP duration, right panel **(E,F)** iSP depth. Top row = left FDI, bottom row = right FDI. Solid line indicates mean difference (bias), dotted lines represent upper and lower 95% limits of agreement.

The results of the Bland and Altman analyses (**Figures 2** and **3**) highlight variability between sessions for some participants, but indicate that there are no systematic differences in the pattern of variability observed. The limits of agreement are wide for both hemispheres, particularly for the depth of the iSP. There are some clear outliers (outside of 95% limits of agreement) and so ICC results are presented with and without these outliers.

The results for the ICCs are shown in **Table 2**. For iSP latency there was moderate to good reliability for the younger adults bilaterally and for the left FDI of the older group (ICC, 0.69–0.91), and poor to moderate reliability for the right FDI of the older group. Removal of outliers improved reliability to a small extent. For iSP duration there was moderate to good reliability for both groups bilaterally, with all ICC values > 0.6. Removal of outliers made a noticeable difference only for the right FDI of the younger group, with ICC values improving from moderate to good. For iSP depth there was poor reliability for both groups for the left FDI when the first two sessions were considered (ICC < 0.4) but moderate reliability if all four sessions were considered (ICC, 0.63–0.65). The ICC values improved if the outliers were removed from the younger group but there were no clear outliers for the older group (**Figure 3**). For the right FDI the reliability of iSP depth was good for the older group and moderate for the younger group, which improved to good with removal of the outliers.

**Table 2:** Intraclass correlation coefficients.

	Younger group				Older group			
	Left FDI		Right FDI		Left FDI		Right FDI	
	All included	Outliers removed	All included	Outliers removed	All included	Outliers removed	All included	Outliers removed
iSP latency								
Two sessions	0.80	0.93	0.69	0.72	0.75	0.92	0.58	0.67
Four sessions	0.89	0.94	0.79	0.80	0.91	0.96	0.79	0.74
iSP duration								
Two sessions	0.90	0.91	0.68	0.86	0.77	–	0.90	–
Four sessions	0.91	0.93	0.73	0.95	0.92	–	0.97	–
iSP depth								
Two sessions	-0.01	0.54	0.62	0.81	-0.40	–	0.86	–
Four sessions	0.63	0.75	0.74	0.83	0.65	–	0.92	–

*– indicates no outliers to be removed. ICC values > 0.8 represent good reliability, 0.6–0.8 represent moderate reliability, and values < 0.6 represent poor reliability*.

### Comparison between Sessions

For the younger group, there was no difference in baseline EMG activity across sessions for either hand (*p* > 0.3), indicating no difference in the level of voluntary contraction. For iSP duration, the ANOVA revealed a significant effect of session for left FDI (*F*_2.1,35.0_ = 3.873, *p* = 0.03) as duration was less for session 2 than all other sessions (*p* < 0.05 with Bonferroni correction), but there were no differences across sessions for the right FDI (Friedman test; *p* = 0.22). For iSP depth and latency there were no differences across sessions for either hand (*p* > 0.2).

For the older group, the Friedman tests showed no differences in baseline EMG activity across sessions for the left FDI (*p* = 0.31), but a significant difference for the right FDI (*p* = 0.03). EMG activity tended to be lower for session 1 than sessions 3 and 4, and for session 2 compared with 3, but these were not significant with Bonferroni correction. There were no differences across the four sessions for either hand for iSP duration (*p* > 0.5), depth (*p* > 0.09), or latency (*p* > 0.09).

## Discussion

This study examined the intersession reliability of TCI using the assessment of iSP latency, duration and depth. The Bland and Altman plots indicate wide variation in limits of agreement for both groups and all measures (**Figures 2** and **3**), but no clear pattern to the variability is observed. The ICCs indicate moderate to good reliability for iSP duration for both groups (**Table 2**), suggesting consistency in this measure. However, iSP depth tended to be less reliable, particularly for the assessment of the first two sessions for the left FDI. There were clear outliers and removal of these participants improved the reliability substantially.

To our knowledge this is the first study to examine the reliability of the iSP as a marker of TCI in healthy younger and older adults. De Gennaro et al. (2003) examined reliability of interhemispheric inhibition using MEP amplitude (paired pulse technique) and demonstrated poor test–retest reproducibility. The duration of the iSP would appear to show better reproducibility, although it is unclear why this would be the case. It may lie in the differences between the two techniques, as the paired pulse (dual coil) technique involves the reduction of MEP amplitude by the conditioning stimulus applied to the opposite hemisphere, whereas the iSP measure used in the present study involves a suppression of voluntary muscle activity. MEP amplitude is inherently variable itself (Amassian et al., 1989), which may lead to an increase in observed variability. The iSP measure is thought to better reflect the control of voluntary movement than the paired pulse technique (Beaule et al., 2012). However, there are possible variations in testing from session to session that could affect reliability of the iSP also. Variation in TMS coil placement and orientation could affect the neurons stimulated and therefore the transcallosal effect, but this was not possible to assess specifically in the current study. Perhaps the most likely issue affecting reproducibility of the iSP is that the amount of voluntary muscle activity could be inconsistent. In the current study the baseline EMG activity did not significantly differ across sessions for either group from the left FDI. However, for the older adults only there was an effect of session for the right FDI activity, with a tendency for activity to increase over the sessions. Subtle changes in baseline EMG may account for some of the between-session variability, particularly for the depth of inhibition which appeared to be substantially less reliable than iSP duration. However, to our knowledge only one study has tested the effect of varying the magnitude of the contraction, finding no effect on the time course of the iSP or the depth of inhibition when expressed as a percentage of the baseline EMG level (Ferbert et al., 1992). Therefore, it would appear unlikely that variation in muscle activity is solely responsible for variation between sessions.

Although between session reliability of iSP duration was found to be moderate to good when assessed using ICCs, the duration from left FDI was significantly lower in session 2 than the other sessions for the younger group and the Bland and Altman plots revealed a wide range of differences between sessions for both hemispheres (**Figures 2** and **3**). Similarly, although there was no significant effect of session on the iSP depth, the Bland and Altman plots reveal wide limits of agreement indicating poor reproducibility (**Figures 2** and **3**). These apparent differences in findings further highlight the importance of using multiple methods to investigate reliability; an ANOVA may show no significant difference, but does not provide any assessment of whether pairs of data are in agreement; ICCs reflect the degree of correspondence and agreement between pairs of measurements (Portney and Watkins, 2000); and a Bland and Altman analysis provides a visual representation of the agreement between measures, allowing patterns in the spread of differences to be realized (Bland and Altman, 1986; Portney and Watkins, 2000).

Previous studies have indicated a change in TCI with aging, with reduced duration and area of the silent period for older adults (McGregor et al., 2011; Davidson and Tremblay, 2013; Coppi et al., 2014), which physical activity may ameliorate (McGregor et al., 2011). The present study showed no clear difference in reliability between younger and older groups suggesting that reproducibility of these measures is not dependent on age. Instead, a difference between hands was observed. There were reduced ICC values for the right hand compared with the left for iSP duration and latency. Coppi et al. (2014) reported iSP duration to be longer for the left hand than the right, but this is the first study to demonstrate that the reliability may differ also. It is unclear why this was the case, but all participants in the current study were right handed and we therefore speculate that the non-dominant M1 may exert a more variable inhibitory influence on the dominant M1 than vice versa. However, this is inconsistent with the findings for iSP depth which was more reliable for the right hand than the left. The non-dominant hand may be less consistent in its force production, which may account for reduced consistency with this measure. Future investigation is required in order to gain an understanding of the variables that contribute to between-session variability of the iSP.

Davidson and Tremblay (2013) used a stimulation intensity of 120% resting motor threshold, which is likely to be lower than the 80% MSO used in the present study and therefore could have influenced the iSP measure (Meyer et al., 1995; Chen et al., 2003). We chose a higher intensity, which is consistent with several other studies (Chen et al., 2003; Trompetto et al., 2004; Stinear et al., 2008, 2015; Spagnolo et al., 2013), in an attempt to ensure maximal activation of transcallosal neurons. Previous studies have demonstrated that the latency and duration of the iSP plateaus at ∼75% MSO (Meyer et al., 1995; Chen et al., 2003) and Ferbert et al. (1992) demonstrated that stimulation intensity affected the depth of inhibition, but not the time-course of the silent period. Therefore, the stimulation intensity chosen may not have been appropriate for the assessment of iSP depth and we cannot discount the possibility that the reliability may be improved if the stimulation intensity was optimized for each participant. Additionally, there are different methods for determining the criterion EMG level at which the onset of the silent period is detected. We chose to use 75% of the baseline RMS EMG, but others have used the baseline level itself (Davidson and Tremblay, 2013; Spagnolo et al., 2013; Stinear et al., 2015), a reduction of one-third (Stinear et al., 2008), 50% (Shimizu et al., 2002) or 1 or 2 standard deviation (Chen et al., 2003; Fling et al., 2011). It is possible that the reproducibility may be affected by the criterion used.

Although iSP duration would appear to be the most reproducible of the measures tested here, if used in a longitudinal study as a marker of change in neurophysiological function, e.g., after stroke, then a large change in duration (∼10 ms) may be required in order to be clinically meaningful. There was also a large inter-individual range in all of the iSP measures (**Figures 2** and **3**). We did not test any other neurophysiological or functional parameters for these participants so cannot demonstrate whether this variability can be attributed to differences in cortical excitability or bimanual motor performance between people. However, previous studies have demonstrated that reduced iSP area and delayed onset are associated with reduced motor performance in healthy young and older adults (Davidson and Tremblay, 2013; Coppi et al., 2014). Additionally, we utilized two different methods for obtaining the voluntary contraction of the FDI, as some participants did not show consistent EMG activity with one method. It is possible that this difference could have contributed to the variability observed.

Overall these results indicate that iSP duration can be a reliable measure of TCI across sessions for healthy adults, but there is variation between participants and a large change may be required for longitudinal studies to be meaningful. The depth of the iSP appears to be less reliable, particularly for the non-dominant hand, and therefore changes should be interpreted with caution. Further research is needed to probe for mechanisms underlying variability in the iSP measures and this study should be repeated in adults with neurological conditions, such as stroke, to determine whether similar results are found.

## Ethics Statement

All participants gave written informed consent in accordance with the Declaration of Helsinki. The protocol was approved by the King’s College London Research Ethics Committee.

## Author Contributions

MF was involved in the conception and design of the experiment as well as collecting, analyzing and interpreting the data. MF additionally led the writing and critical revision of the article. DN was involved in the conception of the experiment, interpretation of data and critical revision of the article.

## Conflict of Interest Statement

The authors declare that the research was conducted in the absence of any commercial or financial relationships that could be construed as a potential conflict of interest.
